# ANCUT1, a novel thermoalkaline cutinase from *Aspergillus nidulans* and its application on hydroxycinnamic acids lipophilization

**DOI:** 10.1007/s10529-024-03467-2

**Published:** 2024-02-28

**Authors:** Carolina Peña-Montes, Eva Bermúdez-García, Denise Castro-Ochoa, Fernanda Vega-Pérez, Katia Esqueda-Domínguez, José Augusto Castro-Rodríguez, Augusto González-Canto, Laura Segoviano-Reyes, Arturo Navarro-Ocaña, Amelia Farrés

**Affiliations:** 1 Tecnológico Nacional de México/IT Veracruz, Unidad de Investigación y Desarrollo en Alimentos (UNIDA), Calzada Miguel Angel de Quevedo, 2779. Col. Formando Hogar, Veracruz, México CP 91897; 2 Departamento de Alimentos y Biotecnología, Facultad de Química, Universidad Nacional Autónoma de México (UNAM), Ciudad Universitaria, CP 04510 Ciudad de México, Mexico; 3 Tecnológico Nacional de México/IT Mochis, Juan de Dios Batiz y 20 de Noviembre, CP 81259 Los Mochis, Sinaloa Mexico; 4 Unidad de Medicina Experimental, Facultad de Medicina, Universidad Nacional Autónoma de México (UNAM), Hospital General de México, Dr. Balmis, 148, CP 06726 Ciudad de México, Mexico

**Keywords:** Cutinases, Thermoalkaline, Purification, Lipophilization, *Aspergillus nidulans*

## Abstract

One of the four cutinases encoded in the *Aspergillus nidulans* genome, ANCUT1, is described here. Culture conditions were evaluated, and it was found that this enzyme is produced only when cutin is present in the culture medium, unlike the previously described ANCUT2, with which it shares 62% amino acid identity. The differences between them include the fact that ANCUT1 is a smaller enzyme, with experimental molecular weight and pI values of 22 kDa and 6, respectively. It shows maximum activity at pH 9 and 60 °C under assayed conditions and retains more than 60% of activity after incubation for 1 h at 60 °C in a wide range of pH values (6–10) after incubations of 1 or 3 h. It has a higher activity towards medium-chain esters and can modify long-chain length hydroxylated fatty acids constituting cutin. Its substrate specificity properties allow the lipophilization of alkyl coumarates, valuable antioxidants and its thermoalkaline behavior, which competes favorably with other fungal cutinases, suggests it may be useful in many more applications.

**Supplementary Information:**

The online version contains supplementary material available at 10.1007/s10529-024-03467-2.

## Introduction

Cutinases (EC 3.1.1.74) are enzymes classified as carboxylesterases. This group includes a diverse group of hydrolases, which catalyze the cleavage of the ester bond in a variety of substrates. Cutinases are named for their hydrolytic action on the ester bonds linking the fatty acids that make up cutin, a structural component of the cuticle that covers the aerial parts of plants and whose primary function is to serve as a protective barrier against physical, chemical, and biological environmental factors, including pathogens (Kolattukudy et al. 1981; Arya and Cohen 2022). The production of natural cutinases is mainly related to phytopathogenic fungi; however, they are also found in other microorganisms such as bacteria and in plants that can degrade the cutin barrier of the stigma to promote the fertilization process (Liang and Zou 2023).

In addition to natural polyesters, cutinases hydrolyze other related substrates such as lipids, waxes and various synthetic esters. They can also catalyze biosynthesis reactions such as esterification and transesterification (Liang and Zou 2023). Due to their versatility, cutinases have been proposed for a wide range of applications in industrial processes, including food, textile, detergent, agricultural, chemical and environmental industries (Chen et al. 2013; Dutta et al. 2009). Particularly applications in the environmental area have stood out because cutinases exhibit hydrolytic activity towards a variety of soluble synthetic esters, synthetic fibers (polyethylene terephthalate fibers) and other polyesters (polycaprolactone, polylactide) that are part of plastics, which are a major source of environmental pollution (Kim et al. 2005; Maeda et al. 2005; Wei and Zimmermann 2017; Wei et al. 2020).

On the other hand, there is also interest in the potential applications of these enzymes in the synthesis of fine chemicals by biocatalysis in non-aqueous media to produce numerous high-value compounds (Bornscheuer 2002; Jeong and Park 2008). There are numerous reports on the enzymatic esterification of hydroxycinnamic acids (HCAs) using carboxylesterases, especially for alkyl ferulates (Grajales-Hernández et al. 2021; Sharma et al. 2014; Tsuchiyama et al. 2007; Vafiadi et al. 2008; Vega-Rodríguez et al. 2021).

Hydroxycinnamic acids (such as ferulic, caffeic, sinapic and p-coumaric) are a group of compounds that are very abundant in foods and have gained increasing interest in the health field because they are known to be potent antioxidants (Teixeira et al. 2013). HCAs are incorporated into foods, cosmetics and drugs to prevent oxidation of substrates such as low-density lipoproteins (LDL) or “bad cholesterol” (Vafiadi et al. 2008). However, due to their low solubility in non-aqueous phases, their application in hydrophobic media is limited. To improve their solubility properties and even enhance their properties, some authors have studied the modification of their structures through chemical and enzymatic reactions involving the addition of aliphatic groups to increase the hydrophobicity of the molecule (lipophilization) (Schär and Nyström, 2015).

*Aspergillus nidulans* is a saprophytic filamentous fungus used as a genetic model organism that can conduct many extracellular enzymatic activities, e.g., *A. nidulans* has been reported to produce several enzymes that hydrolyze ester bonds (Bermúdez-García et al. 2017; Machado and Castro-Prado 2001; Mayordomo et al. 2000; Peña-Montes et al. 2008). The information in the available genome sequence database of *A. nidulans* shows the presence of four different putative cutinases (Galagan et al. 2005). We have reported the purification and characterization of the cutinase ANCUT2 produced by *A. nidulans* induced by olive oil, some triacylglycerides and fatty acids (Bermúdez-García et al. 2017; Castro-Ochoa et al. 2012). Additionally, we have characterized the cutinolytic system and its regulation in *A. nidulans* (Bermúdez-García et al. 2019).

This work describes the purification and biochemical properties of the 22 kDa cutinase (ANCUT1) isolated from *A. nidulans* induced by cutin. Besides, its potential for modification of *p*-coumaric acids is also demonstrated.

## Materials and methods

**Microorganism and maintenance***. A. nidulans* PW1 (*biA1, argB2, methG1, veA1*), a nonpathogenic arginine auxotroph, can be obtained from FGSC as described (Bermúdez-García et al. 2019). It was maintained in silica gel stocks. Conidial suspensions were generated after growth on minimal medium agar with the appropriate supplements, as Käfer et al. (1970) described, and the pH was adjusted to 6.5. Fungal spores were produced on minimal nitrate agar plates, as previously described by Bermúdez-García et al. (2019).

### Optimized medium for ANCUT1 production

First, inducer and optimal inducer concentration were obtained. The minimal medium was prepared as described by Käfer et al. (1970), replacing the carbon source (glucose 1% w/v) with apple cutin, apple cuticle, tomato cuticle, and potato cuticle at a concentration of 0.4% w/v as inducer, and carbon source. To determine the optimal cutin induction concentration, different concentrations of optimal cutin inducer in the range of 0.1–0.5% with an interval of 0.1 were added to the minimal medium (minimal cuticle medium, MCM). Then, an additional carbon sources effect on the MCM medium’s cuticle was tested. For this purpose, the additional carbon source was varied using glucose (0.1 and 1%), glycerol (0.1, 0.5 and 1%), saccharose (0.1%) and starch (0.1%).

Evaluation of the effect of nitrogen source was done by using MCM without 20X nitrate salts and supplemented with the following nitrogen sources (0.06%): inorganic nitrogen source (NaNO_3_, KNO_3_, NH_4_NO_3_, (NH_4_)_2_SO_4_ and urea) and organic nitrogen source (yeast extract and Bacto Peptone).

Each media was sterilized and inoculated with 1 × 10^6^ spore/mL of *Aspergillus nidulans*.

### Isolation of cuticle and cutin

Mature Golden delicious apple (*Malus domestica*), ripe tomatoes (*Lycopersicum esculentum*) and potatoes (*Solanum tuberosum*) were purchased from the local market. The isolation was done according to Kolattukudy et al. (1981) with the following modifications.

### Cuticle extraction

Potatoes, tomatoes and apples were peeled using a knife, and the peel was added to a boiling aqueous solution containing oxalic acid and ammonium oxalate for 15 min or until fully devoid of pulp. Isolated cuticular layers were washed with distilled water, filtered and oven-dried (50 °C). Finally, they were ground to a fine powder and stored in a sealed container at room temperature.

### Preparation of apple cutin

The cuticular layers collected were thoroughly washed with water and mixed with an excess of a 2:1 mixture of chloroform and methanol (20 ml/g wet weight) and then subjected to Soxhlet extraction with chloroform for 6 h. After air drying, the resulting powder was treated with cellulase and pectinase in 0.05 M acetate buffer at pH 4.0 for 14 h at room temperature with agitation. Soxhlet extraction with chloroform and enzymatic hydrolysis was repeated. The solid material collected was washed and dried at 50 °C.

### Culture conditions

All experiments were conducted in 250 mL Erlenmeyer flasks containing 50 mL of an appropriate sterile culture medium, inoculated with 1 × 10^6^ spores/mL. The flasks were incubated in a rotary shaker at 300 rpm (New Brunswick Scientific Innova 40) at 37 °C for 144 h.

## Enzymatic assays

### Carboxylesterase activity assays

Esterase activity against *p*-nitrophenyl acetate (*p*-NPA) was determined in microplates (170 µL of 50 mM phosphate buffer pH 7.2, 20 µL of stock 1 mM de *p*-NPA and 10 µL of enzyme). The substrate hydrolysis by reaction media was evaluated, replacing the enzyme with buffer. All enzyme assays were achieved in triplicate, and activity was quantified by measuring absorbance at 420 nm. One unit of activity was described as the amount of enzyme required to catalyze the conversion of 1 µmol of *p*-NPA to *p*-nitrophenol (*p*-NP) per minute under the assayed conditions.

A standard curve was used to estimate the formation of *p*-NP, and it was prepared in ethanol with *p*-NP concentrations ranging from 25 to 200 µmol and a molar extinction coefficient of 4900 cm^–1^ M^–1^ was obtained. The reaction kinetics was followed for 10 min at RT. A predetermined protocol in the software Gen5 1.10 provided with the Epoch spectrophotometer (BioTeK, Vermont, USA) was used.

### Qualitative carboxylesterase activity assay

Esterase activity was monitored in microplates according to the method described by Bermúdez et al. (2017).

### Protein concentration

The protein concentration was determined using the Bradford method (1976) with a commercial protein assay kit following the provider’s instructions (Bio-Rad Laboratories, Richmond, California, USA). We used bovine serum albumin as a protein standard to construct the calibration curve.

A defined protocol for protein quantification in the Gen5 1.10 spectrophotometer software was applied (BioTek, Vermont, USA). The reaction mixture containing 160 µL of enzyme or water as blank with 40 µL of the Bradford reagent was incubated at RT for 5 min in a microplate. The absorbance at 595 nm was registered.

### SDS-PAGE and zymograms

SDS–PAGE was carried out using 14% acrylamide gels, as Laemmli (1970) described. The visualization of proteins was done by staining the gels with silver according to Chevallet’s method (Chevallet et al. 2006). The molecular weight of the proteins was determined by comparing their mobility to that of a low-range protein marker containing a mixture of six proteins ranging in size from 14 to 97 kDa (Bio-Rad Laboratories, Richmond, California, USA).

After the protein samples were separated by SDS–PAGE, esterase activity was detected using zymography. The enzymes were renatured by washing the acrylamide gel with 0.05 M phosphate buffer (pH 7.2) for 30 min with constant RT agitation. A second wash was performed in the same phosphate buffer containing 5% Triton X-100 under the same conditions.

Esterase activity was visualized by submerging the gel for 15 min in RT in buffer A (50 mM phosphate buffer pH 7.2, 2.5 mL acetone and 10 mg α-naphthyl acetate) and then in buffer B (50 mM phosphate buffer pH 7.2, 24 µL Triton-X100 and 1.25 mg Fast Red TR base in a final volume of 25 mL). The reaction was incubated until dark red bands indicating CHE activity appeared in the gel.

The gels were documented using the Gel Doc imaging system and analyzed using ImageLab 4.0 software (Bio-Rad Laboratories, Richmond, California, USA).

## Enzyme purification

### Ammonium sulfate precipitation

The fermentation broth was first filtered through Whatman filter paper No. 1 under vacuum pressure to remove the mycelium. Granular ammonium sulfate was added over 30 min until a final concentration of 65% was reached while stirring, according to a standard protocol (Creighton 1997). The precipitated protein was collected by centrifugation at 7650 g for 10 min, dissolved in approximately 1–2 volumes of 50 mM sodium phosphate buffer (pH 7.2) and dialyzed using a membrane with a molecular weight cut-off of 12–14 kDa overnight at 4 °C with constant agitation.

### Ultrafiltration

The dialyzed ANCUT1 protein was concentrated by ultrafiltration with stirring in an Amicon Ultrafiltration Cell using Amicon PLGC membranes (NMWCO 1 and 10 kDa) (Millipore Corporation, Billerica, MA, USA).

### Continuous-Elution Electrophoresis (CEDE)

The cutinase-containing extract was purified using the Mini-Prep Cell system (Bio-Rad Laboratories, Richmond, California, USA). The pump flow was 0.1 mL/minute, and the power remained constant at 1 W. A 14% acrylamide resolving gel was loaded with 500 µL of the concentrated enzyme in a loading buffer with β-mercaptoethanol; samples were prepared by boiling for 5 min. Fifty sequential 250 µL fractions were collected. All the fractions were assessed for esterase activity previously described after adding Triton X-100 (5%) and incubating for 24 h at 4 °C. Positive fractions for esterase activity were analyzed on SDS–PAGE stained with silver. Fractions containing the expected molecular weight band of the CEH enzyme were pooled, analyzed by zymography and used for subsequent experiments.

### Protein identification by LC-MS/MS

The cutinase band was excised from an SDS-PAGE gel, and the protein was enzymatically cleaved into small peptide fragments. The resulting mixture was separated and analyzed by LC-MS/MS system at the Proteomics Unit of the Biotechnology Institute, UNAM, as previously reported (Castro-Ochoa et al. 2012). The MS/MS spectra from the enzymatically generated peptides were analyzed manually and by the SEQUEST software (http://fields.scripps.edu/sequest/) and program Matrix science (Mascot Search Result) (http://www.matrixscience.com).

### Bioinformatics analyses

Once the sequences were obtained, comparison and bioinformatic analyses were conducted using the websites http://blast.ncbi.nlm.nih.gov/Blast.cgi and http://www.expasy.org/. The sequences were compared with those reported in the *Aspergillus nidulans* genome database (http://www.broadinstitute.org). The alignment of multiple sequences was carried out in the MultAlin (Corpet 1988) and Clustal W2 (Larkin et al. 2007) software, and for the signal peptide identification, SignalP 4.1 (Petersen et al. 2011) was used. Potential glycosylation sites were predicted with NetGlyc software (Gupta and Brunak 2002).

The structure model was predicted using Modeller (Webb and Sali 2016). The predicted structure was verified with Errat (Colovos and Yeates 1993). The template was selected using the MPI Bioinformatics Toolkit with a New HHpred Server at its core (Gabler et al. 2020; Zimmermann et al. 2018).

## Biochemical characterization

### Effects of temperature on enzyme activity and stability

The optimum temperature for assaying activity was evaluated by incubating the enzymatic reactions at 30–70 °C. Esterase activity was evaluated as described above. Hydrolysis of substrate because of reaction media was assessed, replacing the enzyme with the buffer.

Thermal stability was determined at various temperatures ranging from 30 to 70 °C by incubating 10 µL aliquots of each cutinase for 15, 30 and 60 min. Subsequently, the reaction tubes were cooled to RT, and the residual esterase activities were tested with the assay described above. Residual activity was estimated by comparing the activity to the untreated enzyme (control). All analyses were performed in triplicate.

### Effects of pH on enzyme activity and stability

The effect of pH values ranging from 5 to 11 on the activity of the purified cutinase was estimated. The standard esterase activity assay is described before. Different buffers at 50 mM were used: sodium acetate (pH 5.0), sodium phosphate (pH 6.0 and 7.0), Tris–HCl (pH 8.0 and 9.0) and CAPS (N-cyclohexyl-3-aminopropanesulfonic acid) (pH 10). A control without enzyme was also evaluated at each pH to evaluate the effect of reaction media on substrate hydrolysis.

The pH cutinase stability was evaluated by incubating the enzyme for 1 and 3 h at 4 °C in the buffers. The cutinase activity of the control incubated at 4 °C was considered 100%. Faster substrate hydrolysis without enzyme for the reactions conducted at pH 9 and 10 was observed because absorbance was measured every half-second for 5 min.

### Effects of metal ions and protein inhibitors

The effects of CaCl_2_, KCl, FeCl_3_, MgSO4, CuSO_4_, NaCl and EDTA on the cutinase were tested. Stock solutions of each compound were made at a concentration of 100 mM, and the final concentrations of each metal ion in the reaction mixture were 1 mM and 10 mM. The effects of the cutinase inhibitors, including DMSO, Tween 80 and SDS (0.1% and 1%) on cutinolytic activity were also investigated. Metal ions and protein inhibitors were added to the enzymatic solutions and incubated at 4 °C in 50 mM Tris–HCl pH 9 for 1 h. The activity percentages were determined by comparison with the control mixture, with no metal ion or inhibitor added. All the above tests were conducted in triplicate. Evaluation of substrate hydrolysis because of reaction media was calculated using a control for each compound without enzyme solution.

### Substrate specificity

Substrate specificity was investigated by replacing the *p*-NPA of the reaction described above with the following substrates: *p*-nitrophenyl butyrate (*p*-NPB), *p*-nitrophenyl myristate (*p*-NPM), *p*-nitrophenyl caprylate (*p*-NPC), *p*-nitrophenyl oleate (*p*-NPO), *p*-nitrophenyl decanoate (*p*-NPD), *p*-nitrophenyl laurate (*p*-NPL), *p*-nitrophenyl palmitate (*p*-NPP) and *p*-nitrophenyl stearate (*p*-NPS) in 0.05 M Tris–HCl buffer at pH 9, at 37 °C for 1 h. The cutin hydrolysis reactions were conducted and revealed as described previously by Castro-Ochoa and coworkers (2012). All tests were carried out in triplicate.

### Stability in organic solvents

Evaluation of substrate hydrolysis because the reaction media tested a control without enzyme for each solvent. The stability of the cutinases in acetone, ethanol, isopropanol, hexane and dimethyl sulfoxide was tested. Each cutinase was mixed with 30 and 50% of each organic solvent and was incubated for 24 h at 4 °C at 50 mM Tris–HCl, pH 9. The activities were then determined, considering the mixture without an organic solvent as 100%. All the above-described tests were carried out in triplicate.

### Determination of kinetic parameters

Lineweaver- Burk plots obtained the km and Vmax values using p-NPA as substrate. Several concentrations of cutinase were incubated in 0.5 mM Tris–HCl buffer (pH 9.0) at 37 °C for 30 min. The final *p*-NPA concentrations were 0.5 mM, 0.75 mM, 1 mM, 1.5 mM and 2 mM. The reaction was measured for 5 min.

### Isoelectric focusing and activity staining

Isoelectric focusing (IEF) was performed as described previously (Peña-Montes et al. 2008). Isoelectric points were estimated using an IEF broad-range protein standard (Bio-Rad Laboratories, Richmond, California, USA). The gels were silver-stained, and α- naphthyl acetate was used as the substrate to detect enzyme activity on zymograms.

### Determination of glycosylation sites

Identifying potential glycosylation sites on the cutinases was made utilizing the DIG Glycan Differentiation Kit (Roche, Penzberg, Germany). The principle of this test is the same as that of a Western blot, but it uses different lectins coupled to an antibody. Each lectin recognizes only one type of sugar, so a positive reaction identifies the sugar bound to the target protein. Thus, positive controls must be used for each lectin residue.

### Propyl ricinoleate (PRO) hydrolysis

A reaction containing 10 mg of freeze-dried cutinase, 10 mg of PRO previously dissolved in a mixture of 0.5 mL of potassium phosphate buffer pH 7.5 and 1 mL of hexane was placed in a 6 mL vial. A similar reaction using the CAL-B enzyme (Novozyme) was tested to compare the hydrolysis of an analogous substrate to the hydroxylated long-chain length esters (C18) contained in the cutin polymer. A control reaction without the enzyme was also evaluated. Products were evaluated using the electrospray ionization mass spectrometry (ESI–MS) on a Leco Pegasus III flight time spectrometer. The reactions were placed for three days at 250 rpm and 37 °C. Samples were taken every 24 h to monitor the hydrolysis of the ester and the appearance of 12-hydroxyoctadeca-9-enoic acid. Reaction products were analyzed by TLC using the solvent system hexane: ethyl ether: formic acid (9:1:0.01). When aliphatic alcohols were tested, TLC plates were revealed using a saturated iodine chamber or spraying with 10% H_2_SO_4_. A free radical scavenging assay to test the antioxidant activity of phenols was performed using the stable free radical 1, 1-diphenyl-2- picrylhydrazyl (DPPH) (Xie and Schaich 2014).

### Transesterification of hydroxycinnamic acid

We tested a transesterification reaction between the methyl group of *p*-coumaric acid and butanol. The expected reaction products were a more liposoluble HA (butyl coumarate (BCUM)) and methanol. The freeze-dried ANCUT1 enzyme was used to test the synthesis reaction with the substrate methyl coumarate (MCUM), a derivative of *p*-coumaric acid, also tested with CAL-B. The reactions were carried out for five days in 8 mL vials at 60 °C and 200 rpm. The reaction components were as follows: 6 mg MCUM, 2.5 mL toluene, 5 mL butanol, 50 mg enzyme (lyophilized extract or CAL-B) and 10% w/v 4 Å molecular sieve with particle size 4–8 mm mesh (Sigma-Aldrich). After the end of the reaction time, TLC plates were run to check the formation of BCUM, the solvents were evaporated, and the reaction products were sent for ESI-MS analysis on a Leco Pegasus III time-of-flight spectrometer. The TLC solvent system was hexane: ethyl acetate: formic acid (7:3:0.01 v/v). TLC staining was done with DPPH as described above and ceric ammonium sulfate by preparing a 1% (w/v) solution of cerium (IV) ammonium (CEAS) in 50% phosphoric acid.

## Results and discussion

### Cutinase production

#### Effect of inducer

**Fig. 1:**
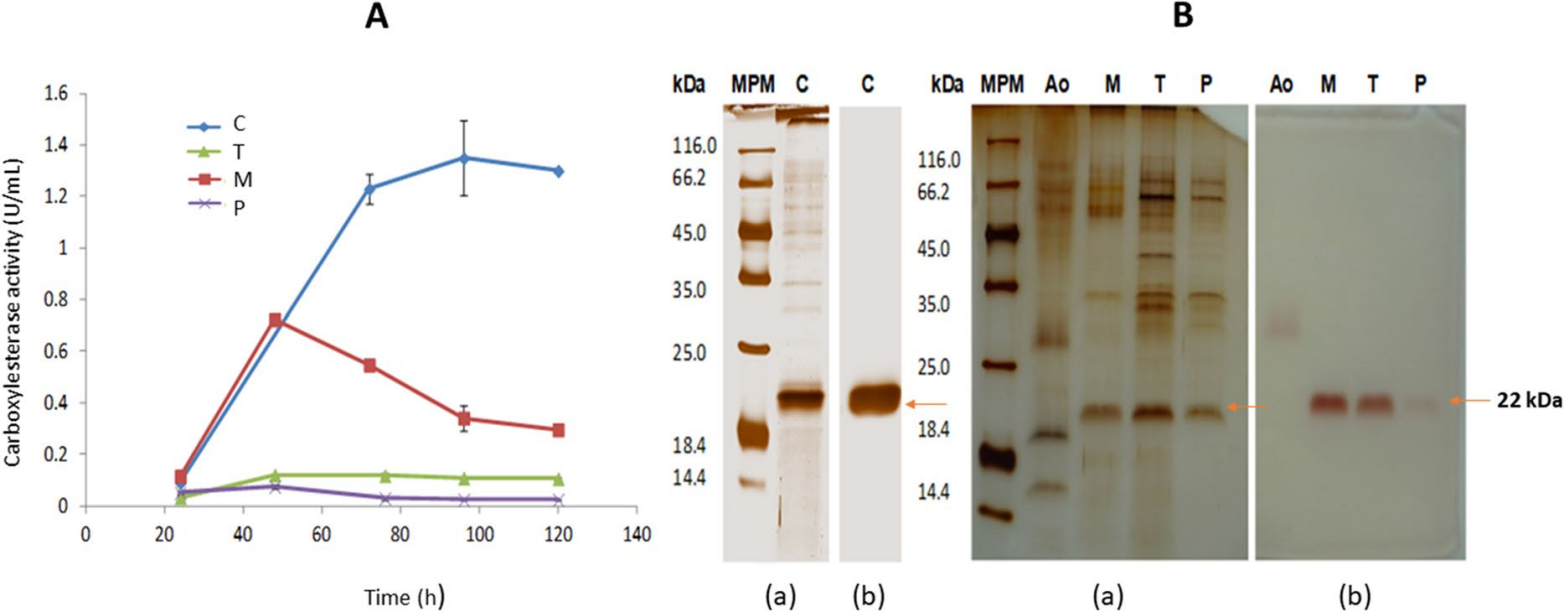
Effect of different inducers on the production of extracellular CEH activity in *A. nidulans* in minimal medium. Panel **A**, Kinetics of CEH activity production. Panel **B**, Protein profiles and in situ activity of extracellular crude extracts on SDS-PAGE, (a) Protein staining with silver nitrate and (b) zymogram using α-naphthyl acetate as substrate. *MPM* molecular protein marker, *C* pure apple cutin, *Ao* olive oil, *T* tomato cuticle, *M* apple cuticle, *P* potato cuticle. Results are the media of three replicates

Adding apple, tomato or potato cuticles to the culture medium induced the production of CEH activity; however, it was lower than that obtained with apple cutin (Fig. 1, Panel A). In the case of cuticles, the apple cuticle was the best inducer for cutinase production (0.722 U/mL), followed by tomato (0.119 U/mL). The lowest production was obtained with the potato cuticle (0.074 U/mL). The cutin source preference for the induction of cutinases varies in each microorganism. Hawthorne et al. (2001) evaluated three sources of cutin (*Cucurbita maxima*, *Cucurbita moschata* and “Granny smith” apple)for cutinase production in two strains of *Fusarium solani* f. sp. One of the strains (SAM410) produced more significant cutinase activity with cutin obtained from *C. moschata*, while the other strain (PGB153) reached a higher activity production with cutin from *C. maxima*.

After growing in apple cutin, the secreted protein pattern by *Aspergillus nidulans*, apple and potato cuticles was very similar when cuticles were used as the sole carbon source and inducer (Fig. 1b). However, with tomato cuticle, a slightly different pattern was produced (more secreted proteins). A majority band with a molecular weight of approximately ≈22 kDa was found in all cases. The zymogram revealed that this ≈22 kDa protein was the only one that showed CEH activity (Fig. 1b, (b)). The protein of ≈22 kDa induced with cutin differs from the enzyme induced by olive oil (Ao) in CEHM (Castro-Ochoa et al. 2012).

**Fig. 2:**
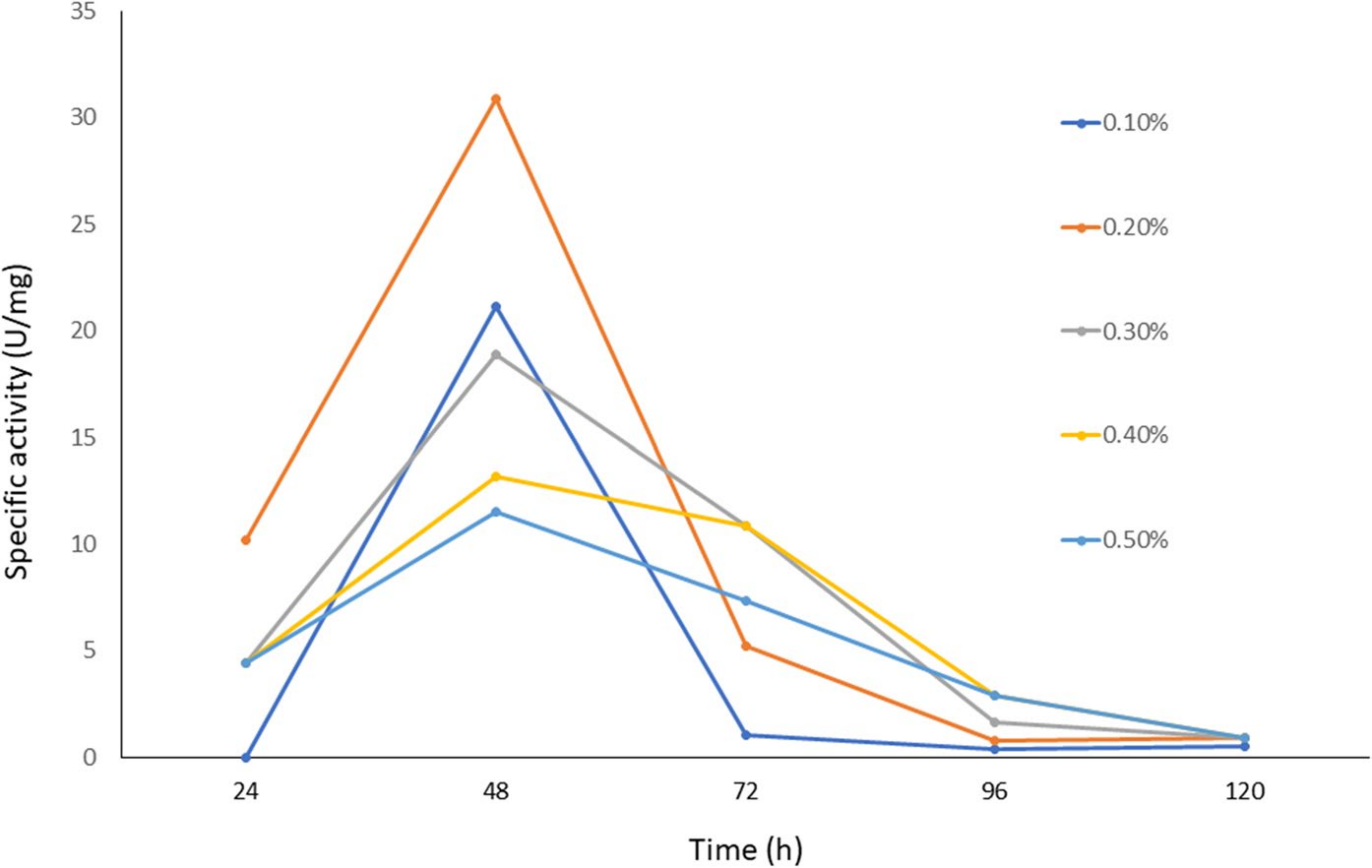
Effect of inducer concentration on the CEH-specific activity of crude extracts of *A. nidulans* in CMM medium. Apple cutin was used as an inducer. Results are the media of three replicates

The effect of inducer concentration was evaluated using golden apple cutin, a source that is the natural substrate for the activity of cutinases. Different cutin concentrations of 0.1–0.5% (w/v) were assessed. Figure 2 shows that the concentration that favored cutinase production was 0.2%; this was observed after 48 h of fermentation when the enzyme reached a specific activity of 30.89 U/mg. The other inducer concentrations evaluated displayed a lower specific activity. It is essential to mention that a higher cutin concentration decreases enzymatic activity, denoting an enzymatic inhibition by the substrate.

#### Effect of carbon source

**Fig. 3:**
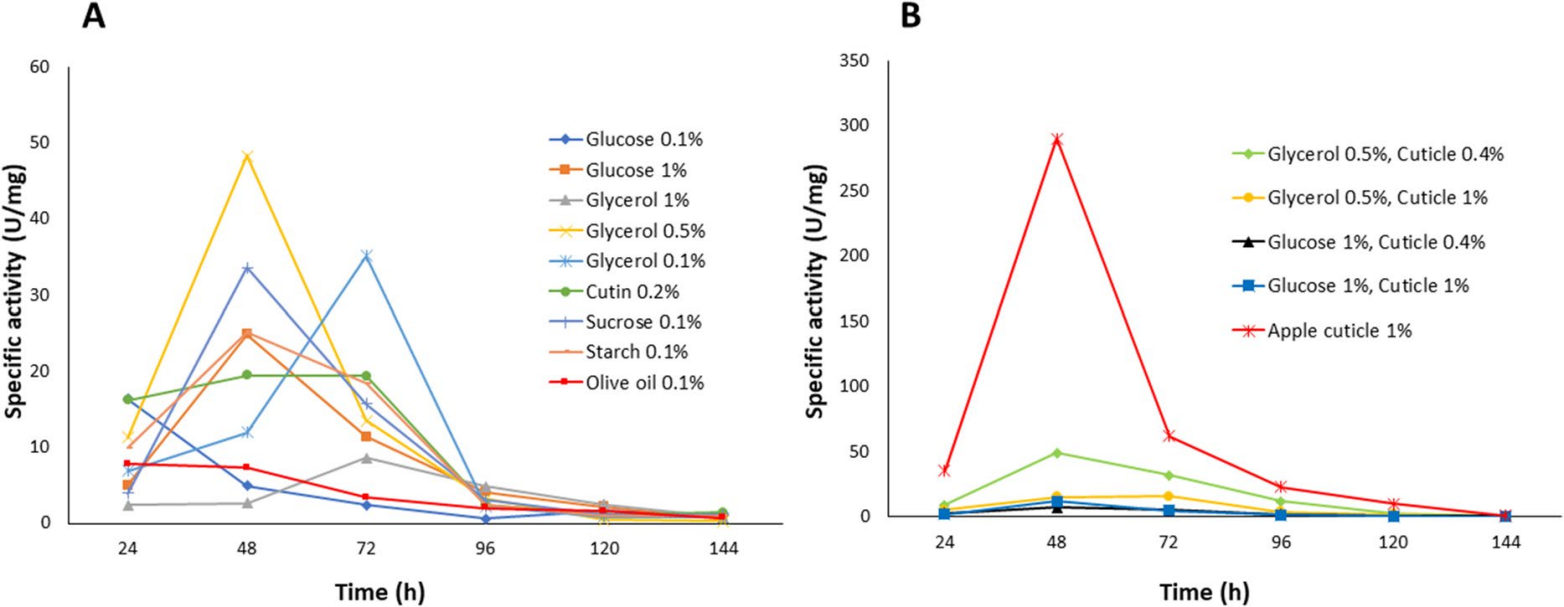
Effect of different carbon source and inducer concentrations on the CEH-specific activity of crude extracts of *A. nidulans* in CMM medium. Panel **A**, the effect of an additional carbon source on apple cuticle in CMM medium. Panel **B** shows the effect of the selected additional carbon source and different inducer concentrations in theCMM medium. Results are the media of three replicates

Each microorganism possesses different metabolic machinery and prefers one carbon source over another (Sánchez and Demain 2002). In fermentation, the carbon source provides the necessary material for the biosynthesis of various cellular macromolecules, such as carbohydrates, proteins, lipids, nucleic acids, etc. Its oxidation provides energy to the cell. Therefore, once the inducer conditions for cutinase production in *A. nidulans* were established, we analyzed the effect of additional carbon sources in the media*.* In addition, catabolic repression by glucose was also investigated. (Fig. 3). The highest specific activity was obtained with glycerol at 0.5% (48.3 U/mg) at 48 h of fermentation, followed by glycerol at 0.1% (35.14 U/mg) at 72 h of fermentation and then 0.1% sucrose (33.64 U/mg) at 48 h of fermentation as shown in Fig. 3a.

After using 0.1% glucose, repression was observed in cutinase production. Maximum CEH activity was reached (16.35 U/mg) after 24 h of fermentation and then decreased. In the case of glucose (1%), it was observed that after 24 h of fermentation, the enzymatic activity was lower (5 U/mg). After 48 h of fermentation, an increase in enzymatic activity was observed (24.90 U/mg). These results indicated that enzyme synthesis does not occur until the carbon source is consumed, a characteristic of the fermentations subject to catabolic repression.

The presence of a carbon source, such as glucose, plays an essential role in the activation of the transcription factor CreA, which intervenes in the catabolic repression of the genus *Aspergillus* in species such as *A. nidulans* and *A. niger* (Castro-Ochoa et al. 2012; Ruijter and Visser 1997; Tanaka and Gomi 2021). Additionally, we considered that the obtained results by using glucose as an additional carbon source could be due to the acidification effects of the culture medium by the metabolism of this carbon source or altered growth. To evaluate this proposal, fermentations of 48 h were carried out with 0.1% and 1% glucose to determine the crude extract’s pH and the mycelium’s dry weight.

In the fermentation with 0.1% glucose, the dry weight of the mycelium did not show a significant difference between 24 h of fermentation and 48 h because the amount of glucose was insufficient for growth. While in the one carried out with 1% glucose, growth was higher at 24 h and continued to increase at 48 h, and the pH became alkaline, probably due to proteolysis. An effect of catabolic repression was observed, as low enzyme production in the presence of glucose was not due to low *A. nidulans* growth or pH inactivation (data not shown).

**Fig. 4:**
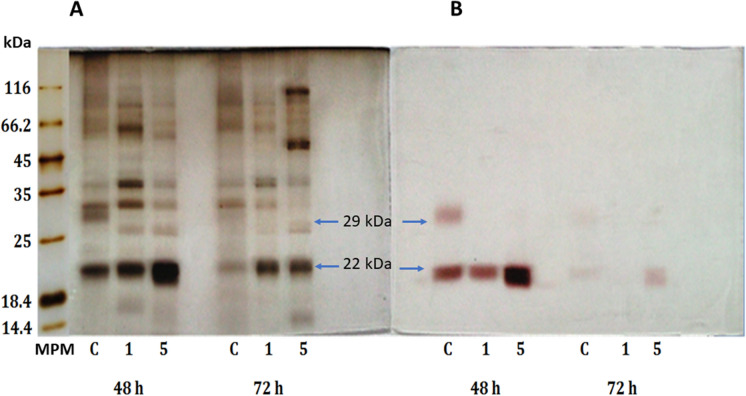
Protein profiles on SDS-PAGE after 48 and 72 h of fermentation of *A. nidulans* in CMM medium. Panel **A**, protein profile after silver staining and panel **B**, zymogram using α-NA as substrate. *MPM* molecular weight marker, *C* CMM medium with only apple cuticle (1%) as carbon source, 1: CMM medium with apple cuticle (0.4%) and glycerol (0.1%), 5: CMM medium with cuticle (0.4%) and glycerol (0.5%). Produced enzymes with CEH activity of 37, 29 and 22 kDa are marked

Once 0.5% glycerol was established as the preferable additional carbon source to the inducer, fermentations were carried out using apple cuticle instead of cutin (0.4% and 1%) (Fig. 3b). Evaluation of the presence of glucose was also included to compare. As expected, the use of glycerol as a preferential carbon source did not significantly affect enzyme activity, and glucose significantly decreased enzyme activity, confirming the repressor effect of this primary substrate on enzyme induction. It is essential to mention that when apple cuticle (1%) was used as inducer and carbon source, activity was significantly increased (290.15 U/mg); however, the protein profile and zymogram analysis displayed two other CEH activity bands (29 and 37 kDa). These two bands correspond to the already reported CEH enzymes of *A. nidulans:* the cutinase ANCUT2 and the protease PrtA (Fig. 4) (Peña-Montes et al. 2008; Castro-Ochoa et al. 2012). In the medium with 0.5% glycerol and 0.4% apple cuticle, only one intense band at ≈22 kDa was observed; the obtained activity was 49.5 U/mg in these conditions. These conditions were set to produce≈22 kDa cutinase, resulting in inducer saving (Fig. 4).

#### Effect of nitrogen source

We evaluated different organic and inorganic nitrogen sources, but they had no significant results for CEH activity. The highest enzyme activity was obtained with potassium nitrate, obtaining an activity of 71 U/mg at 48 h of fermentation. Still, the zymogram analysis displayed the two other CEH enzymes (data not shown) when only the apple cuticle was used as an inducer and carbon source. In the case of sodium nitrate and ammonium nitrate, the highest enzyme activity was obtained at 48 h of fermentation, with values very close (42.89 U/mg and 46.22 U/mg, respectively), observing in zymograms small activity bands corresponding to ANCUT2 and PrtA enzymes. After using ammonium sulfate, the maximum activity occurred at 72 h of fermentation and reached a value of 38.30 U/mg. Urea produced only a maximum enzyme activity of 11.03 U/mg at 72 h of fermentation.

In the case of the evaluation of the two organic nitrogen sources, they showed similar behavior since both produced the cutinase after 48 h of fermentation, and the enzymatic activity values were very close, 19.37 U/mg and 17.24 U/mg. After adding organic nitrogen sources, only the ≈22 kDa band was observed in zymograms (data not shown). However, considering a lower activity than media containing glycerol and apple cuticle without these sources (49.5 U/mg), it was not included in producing the ≈22 kDa enzyme.

### Enzyme purification and identification

Enzyme purification is often a complex process, and several methods are usually applied in sequence to attain a sufficiently high purity level. For cutinase ANCUT1, the purification protocol involves three steps: (NH_4_)_2_SO_4_ precipitation, ultrafiltration and continuous elution electrophoresis. Enzyme activity was recovered after purification for some fractions after using the renaturing agent (Triton X-100). Table 1 shows the yield and purification factors of ANCUT1, 22.94 and 98.96, respectively. The purification process resulted in a 100-fold activity increase.

**Table 1:** Summary of the purification procedure of the cutinase ANCUT1

Stage	Procedure	Fraction Volume (mL)	Final Volume (mL)	Protein concentration (mg/mL)	Total Protein (mg)	Volumetric Activity (U/mL)	Total Volumetric Activity total (U)	Specific Activity (U/mg P)	Yield (%)	Purification Factor (times)
1	Crude Extract	10	10	5.6	56	132.03	1320.29	23.58	100	1
2	(NH_4_)_2_SO_4_, Precipitation Dialysis (14 kDa NMWL)	10	2	0.066	0.132	56.72	113.45	859.45	8.59	36.45
3	Ultrafiltration (1 kDa NMWL)	2	0.4	0.04	0.016	78.97	31.59	1974.33	2.39	83.74
4	Continuous Elution Electrophoresis (CEDE)	0.4	1.5	0.087	0.130	201.96	302.93	2333.22	22.94	98.96

**Fig. 5:**
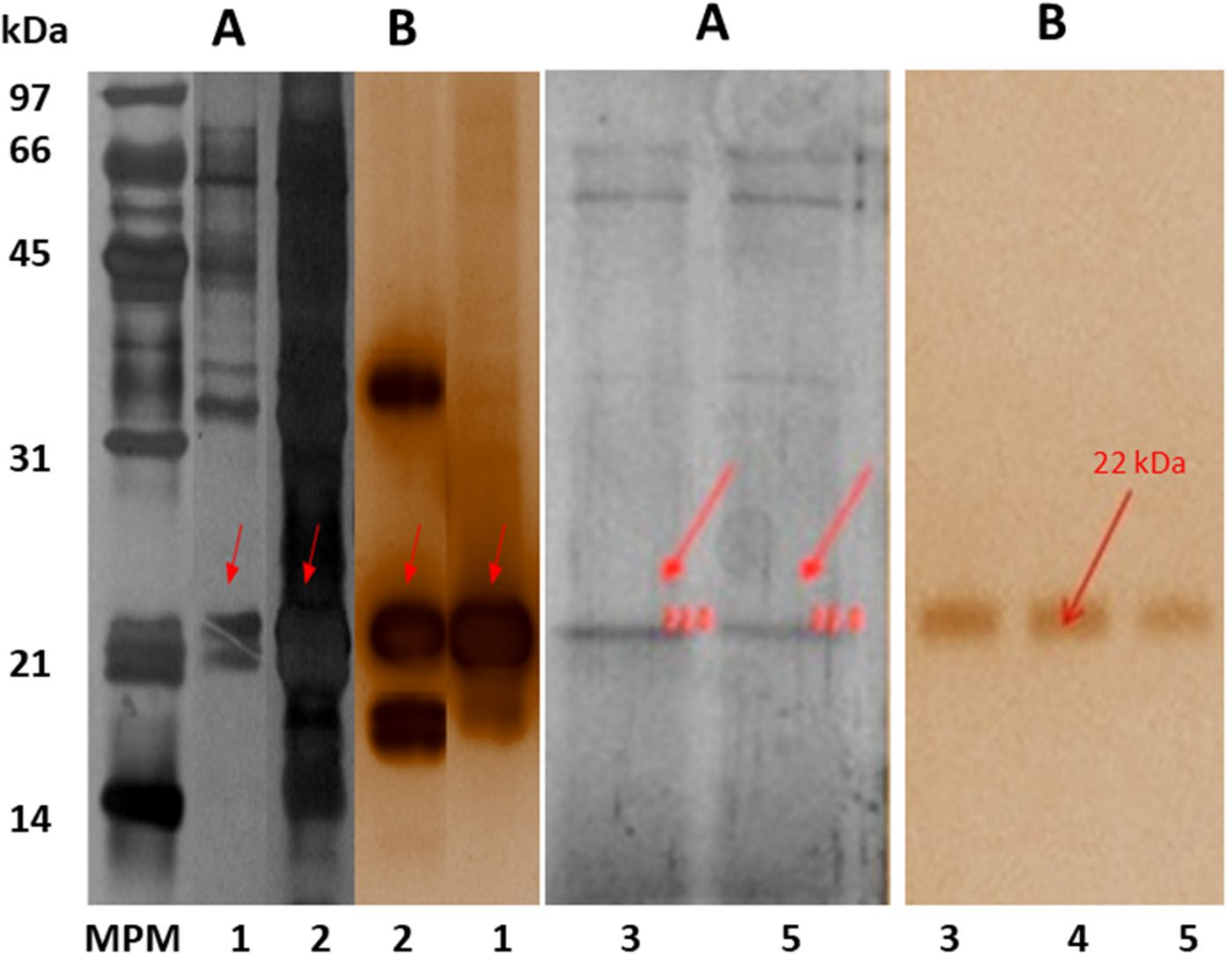
Protein profile on SDS-PAGE from each stage of ANCUT1 purification. Panel **A**, Silver staining. MPM: Bio-Rad Low Range molecular weight, (1): Sample after precipitation and dialysis (stage 1) of *A. nidulans* crude extract of CMM medium after 72 h of fermentation and (2): Ultrafiltrated sample of stage 2 (stage 3), (3) Fraction 33 with CEH activity after CEDE purification (stage 4), (4) Fraction 39 with CEH activity after CEDE purification (stage 4), (5) Fraction 36 with CEH activity after CEDE purification (stage 4). Arrows indicate the 22 kDa enzyme. Panel **B**, Zymogram revealing carboxylic ester hydrolase activity (CEH)

After the enzyme purification procedure, the fractions that showed CEH activity were examined using SDS-PAGE. A ≈22 kDa band was detected in fractions 33–39, pooled, and used for the characterization experiments. Silver staining showed a protein band at the expected molecular weight of ≈22 kDa. Only a single CEH activity band was observed at ≈22 kDa in the zymogram. The image documentation analyses calculated a molecular weight of 22 kDa for this protein band (Fig. 5). This result agrees with the theoretical molecular weight; no *O-* or *N*-glycosylation was detected (data not shown). The observed experimental pI was 6, which agrees with the theoretical value.

**Fig. 6:**
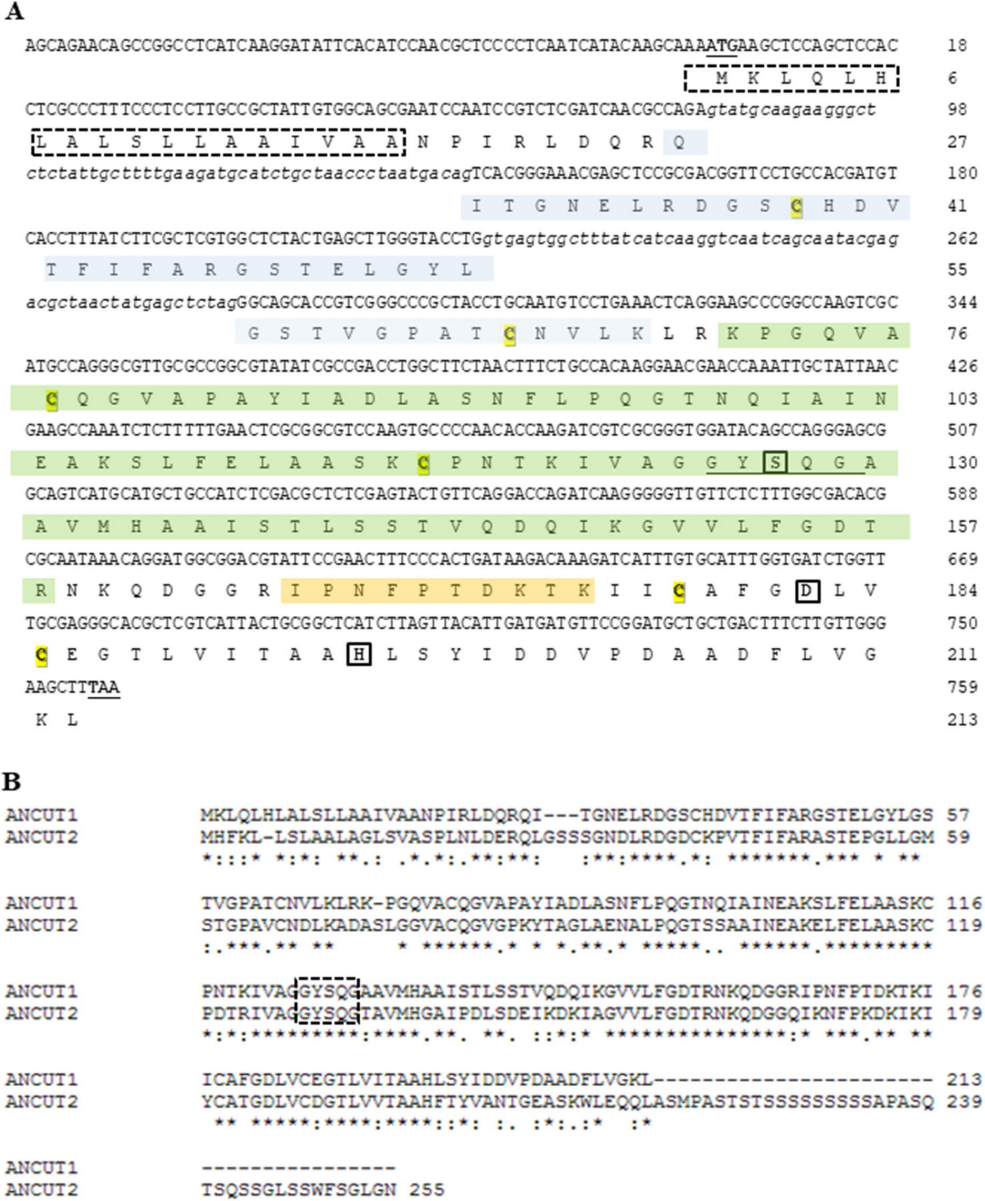
Panel **A**, ANCUT1 genomic and deduced amino acid sequences belonging to the CEH family. The deduced amino acid sequence is presented under the corresponding codons. They are numbered on the right side. The start and stop codons are in bold letters and underlined. The signal sequence is enclosed in a dotted square. The intron sequences are denoted by cursive lowercase letters. The conserved pentapeptide sequence of CEH is underlined. The amino acids from the catalytic triad (Ser-127, Asp-182 and His-195) are enclosed in a bold square. The LC-MS/MS deduced peptide sequences are blue, green and orange. Panel **B**, Comparison of amino acid sequences of ANCUT1 and ANCUT2 cutinases from *A. nidulans*. They are numbered on the right side. The conserved pentapeptide sequence of CEH is enclosed. The six conserved cysteines that can form disulfide bonds are shown in yellow

**Fig. 7:**
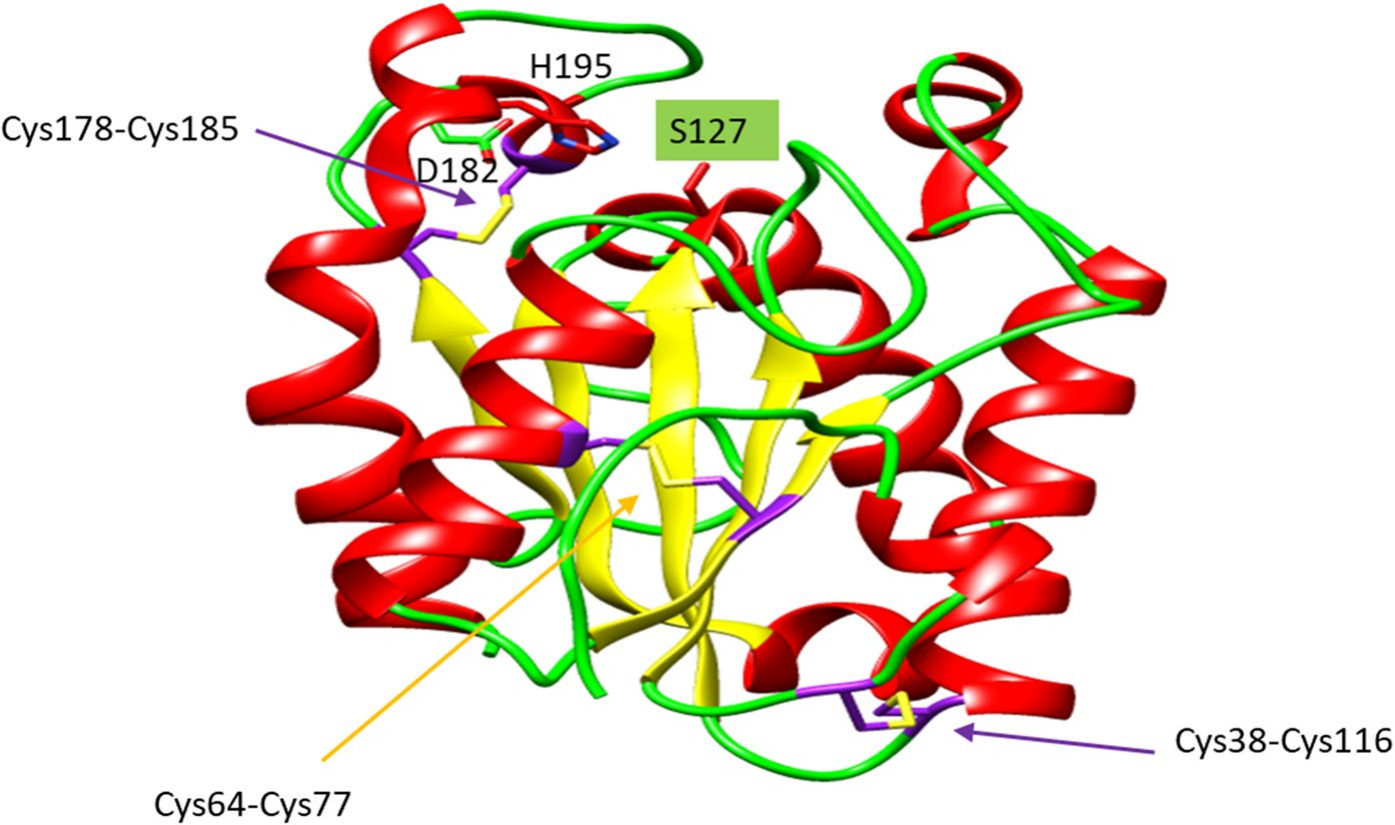
Model of the three-dimensional structure of cutinase ANCUT1 from *A*. *nidulans*. The five α-helices are indicated in red, the central β-sheet is in yellow, and the disulfide bridges are in purple. The disulfide bridge characteristic of *Aspergillus* cutinases, according to Liu et al. (2009), is indicated by the orange arrow. The active site cavity with the catalytic Ser (green) is located at the top of the model

Three peptide sequences were obtained to identify the 22 kDa enzyme. Peptides matched with the translated sequence of the probable cutinase1 gene, *an5309,* according to the genome database of *A. nidulans,* with a theoretical molecular weight of 22 kDa. We denoted it as ANCUT1 (Fig. 6a). It shares 62% of its identity with the previously described ANCUT2, and both have the same pentapeptide consensus sequence (GYSQG), which contains the active serine residue (Fig. 6b) (Bermúdez-García et al. 2017). The serine, aspartic acid, and histidine residues, part of the catalytic triad, are completely conserved. For the novel ANCUT1, the pentapeptide consensus sequence was found at positions 125–129, and Ser127, Asp182, and His195 were identified as the amino acids involved in the active site, which agrees with the obtained structural model, where they are located together at the top of the model with the correct interaction distance (Figs. 6 and 7).

The ANCUT1 amino acid sequence has six cysteine residues that form three disulfide bridges involved in the thermostability. Disulfide bridges can be observed in the predicted structural model (Fig. 7). In Table 2, some thermostable cutinases and the forces and interactions involved are described; as can be observed disulfide bridge play an important role. The disulfide bridge Cys64-Cys77 is characteristic of *Aspergillus* cutinases, according to Liu et al. (2009), and the orange arrow indicates it in Fig. 7. According to the bioinformatics analysis, the closest homologs of ANCUT1 are 5X88 *Malbranchea cinnamomea*, 3QPD *Aspergillus oryzae*, 3DCN *Glomerella cingulata*, 7CY3 *Paraphoma sp*., 4OYL *Humicola insolens* y 7CW1 *Nectria haematococca*. Therefore, these crystallographic structures were used as templates for homology modeling. The structure of *Aspergillus nidulans* cutinase shows an α∕β fold with a central β-sheet of 5 parallel strands, surrounded by 10 α-helices. ANCUT1 possesses an active site formed by the catalytic residues S127, D182, and H195. S127 is found in the pentapeptide conserved in fungal cutinases -GXSXG- located between the β3 sheet and the α-helix 4. The active site cleft comprises 84–92 in helix 3 and 186–205 between helix 9 and 10. It bears an oxyanion hole formed of S49 and Q128. This cutinase contains three disulfide bonds, C38-C116, C64-C77 and C178-C185, which contribute to the thermostability of ANCUT1, as we mention below.

**Table 2:** Characteristics of some thermostable cutinases and the forces and interactions involved

Cutinase	Source organism	Optimum Temperature (°C)	Melting Temperature (°C)	Factors involved in thermostability	References
ANCUT1	*Aspergillus nidulans*	60	N/D	Disulfide bonds, saline bridges, prolines in loop regions	This work
ANCUT2	*Aspergillus nidulans*	60	N/D	Disulfide bonds	Bermúdez et al. (2017)
HiC	*Humicola insolens*	80	62.7	–	Baker et al. (2012)
McCut	*Malbranchea cinnamomea*	45	N/D	Disulfide bonds, prolines in flexible loop regions	Duan et al. (2017)
TtCutopt	*Thielavia terrestris*	50	N/D	Glycosylation	Duan et al. (2019)
IsPETase	*Ideonella sakaiensis*	40	84.7	An strictly conserved disulfide bridge connects the last loop and the C-terminal helix	Brott et al. (2022) and Yoshida et al. (2016)
LCC variant	Metagenome from leaf branch compost	72	94	Insertion of a disulfide bond	Tournier et al (2020)
Cut190 variant	*Saccharomonospora viridis*	70	85.7	Replacement of a Ca^2+^ binding site for a disulfide bond	Numoto et al (2018)
EST119	*Thermobifida alba*	50	N/D	Ca^2+^ dependence	Thumarat et al. (2012)
Cut1	*Thermobifida cellulosilytica*	60	N/D	–	Usman et al. (2023)
Tfu_882	*Thermobifida fusca*	60	N/D	–	Chen et al. (2008)
Tfu_883 TfCut2	*Thermobifida fusca*	60	70	Disulfide bonds, hydrogen bond network, improved aliphatic index	Chen et al. (2008) and Roth et al. (2014)

### Biochemical characterization of the purified cutinases

#### Effect of temperature on activity and stability

**Fig. 8:**
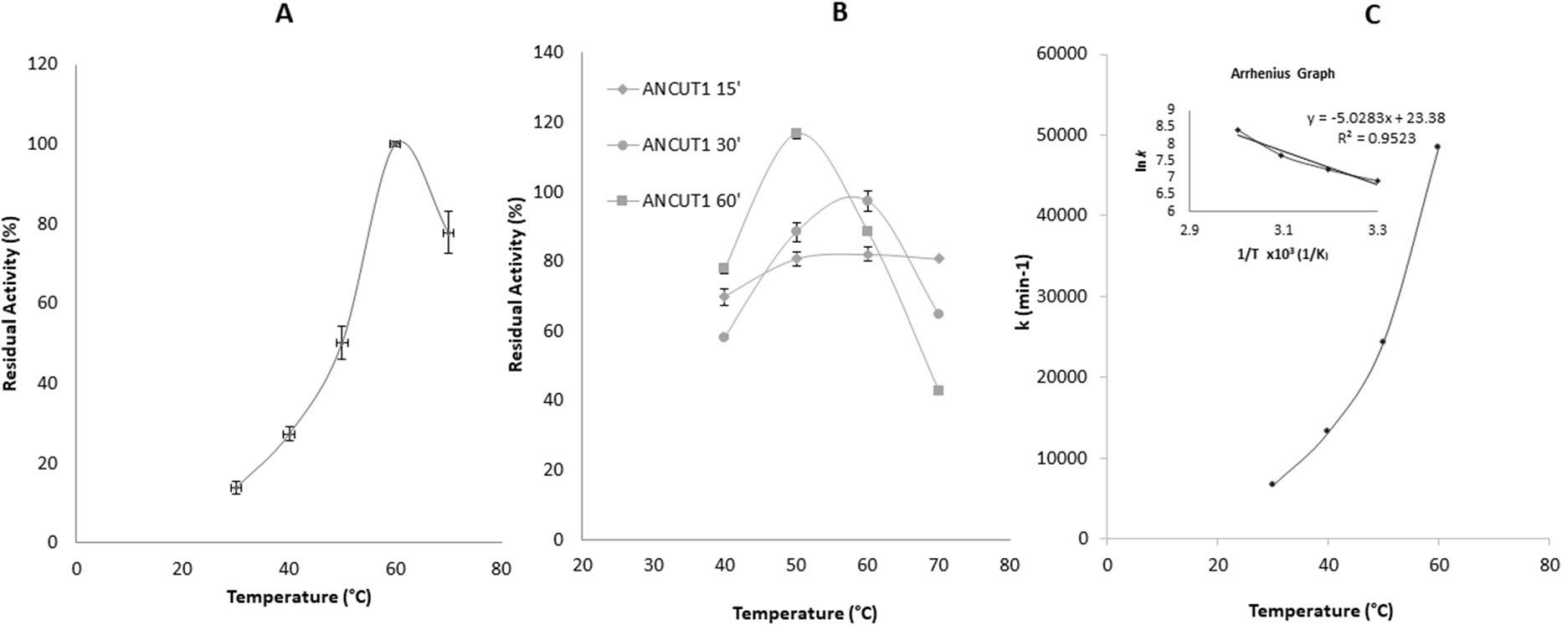
Effect of temperature on activity and stability of ANCUT1. Panel **A**, determination of the optimum temperature for the activity ANCUT1 under assayed conditions. Panel **B** shows the temperature stability of ANCUT1 under different incubation times, and panel **C** shows an Arrhenius plot for the hydrolysis of *p*-NPA by the cutinase ANCUT1

ANCUT1 showed maximum activity at 60 °C and presented significant activity even at 70 °C, showing a residual activity above 70% (Fig. 8a). A similar result was obtained with *T. fusca* cutinase, which has an optimal temperature of 60 °C classified as thermostable (Zhang et al. 2010). It is essential to mention that the enzyme retained more than 80% of its activity after incubation at 60 °C for 1 h, and even it can retain 43% of its activity after one hour at 70 °C (Fig. 8b). The thermostability of ANCUT1 is comparable to the *T. fusca* cutinase, which retains 50% of its activity after incubation at 70 °C for 1 h (Chen et al. 2008), and it is greater than that reported for ANCUT2. The Ea for *p*-NPA, determined by constructing Arrhenius graphs, was 22.834 kcal/mol (Fig. 8c), similar to the value obtained for ANCUT2 (Bermúdez-García et al. 2017) and also comparable to other Ea values reported for thermostable enzymes isolated from thermophilic microorganisms and even from *A. nidulans,* as its protease PrtA has an Ea value of 65.9 kcal/mol) (Peña-Montes et al. 2008; Takao et al. 2000; Thomsen and Nidetzky 2008).

#### Effect of pH on enzyme activity and stability

**Fig. 9:**
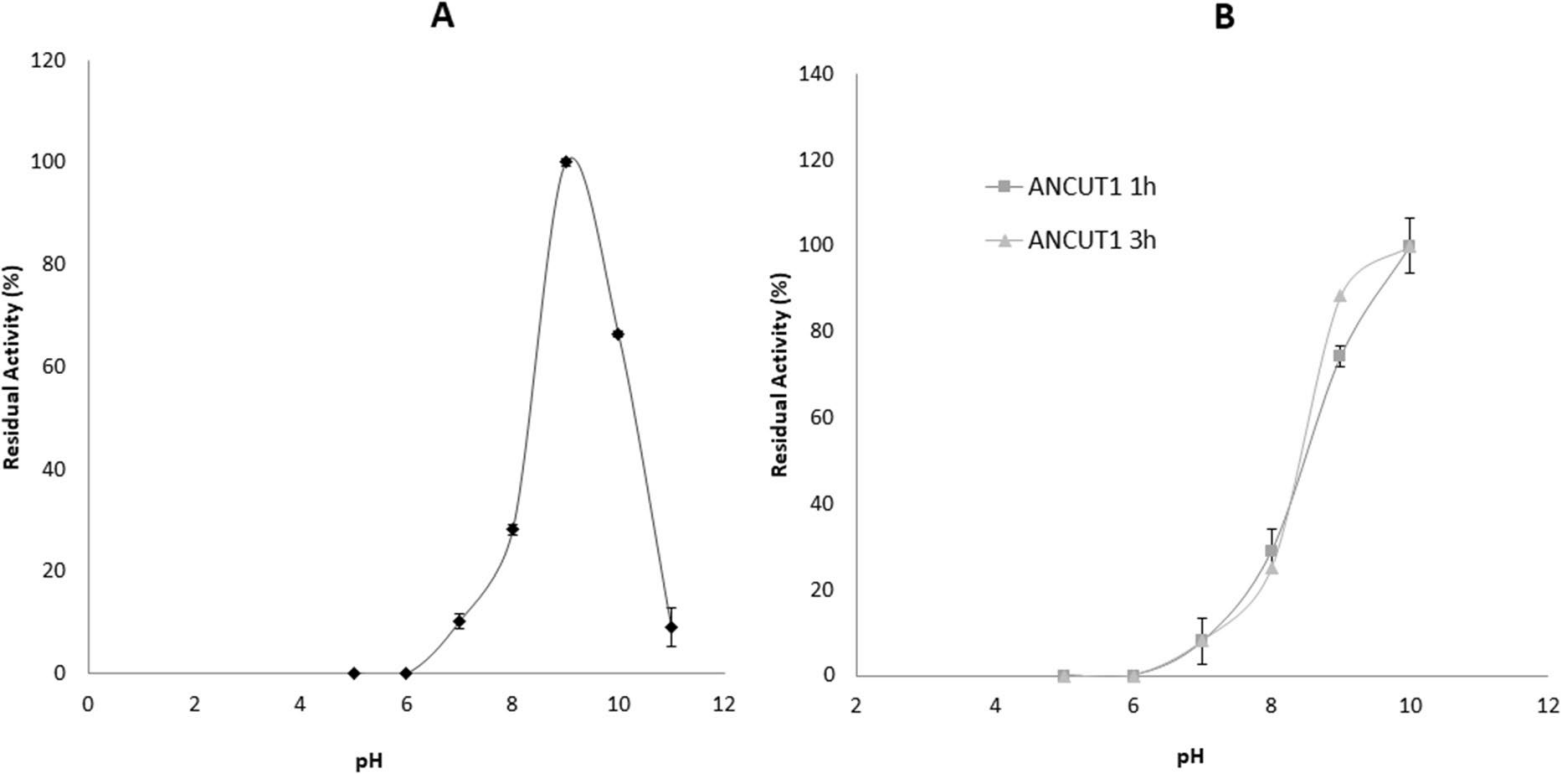
Effect of pH on activity and stability of ANCUT1. Panel **A** shows the effect of pH on the activity of ANCUT1, and panel **B** shows the effect of pH on the stability of ANCUT1 under different incubation times

The optimum pH for ANCUT1 under the assayed conditions is 9, indicating an alkaline cutinase. Other cutinases, such as those from *Fusarium solani* pisi, *A. oryzae* and *T. fusca,* are also alkaline (Chen et al. 2010; Liu et al. 2009; Seman et al. 2014). The pure enzyme retains its activity after 3 h at pH 10 above 85%, but its activity decreases to below 50%. It is still observed that the enzyme has no activity at pH values of 5 and 6, thus confirming the alkaline nature of cutinase ANCUT1 (Fig. 9). Indeed, ANCUT1 is a thermo-alkaline enzyme. Most of the cutinases that have been previously characterized are most active at alkaline pH values (Liu et al. 2009; Maeda et al. 2005; Speranza et al. 2011), but they exhibit maximum activity at 37 °C. Few cutinases have been reported as thermo-alkaline enzymes (Bermúdez-García et al. 2017; Castro-Ochoa et al. 2012). ANCUT2 has six cysteines that form three disulfide bonds that could confer enzyme thermostability; this is also true for ANCUT1, which has six cysteines (Castro-Ochoa et al. 2012).

### Substrate specificity

**Fig. 10:**
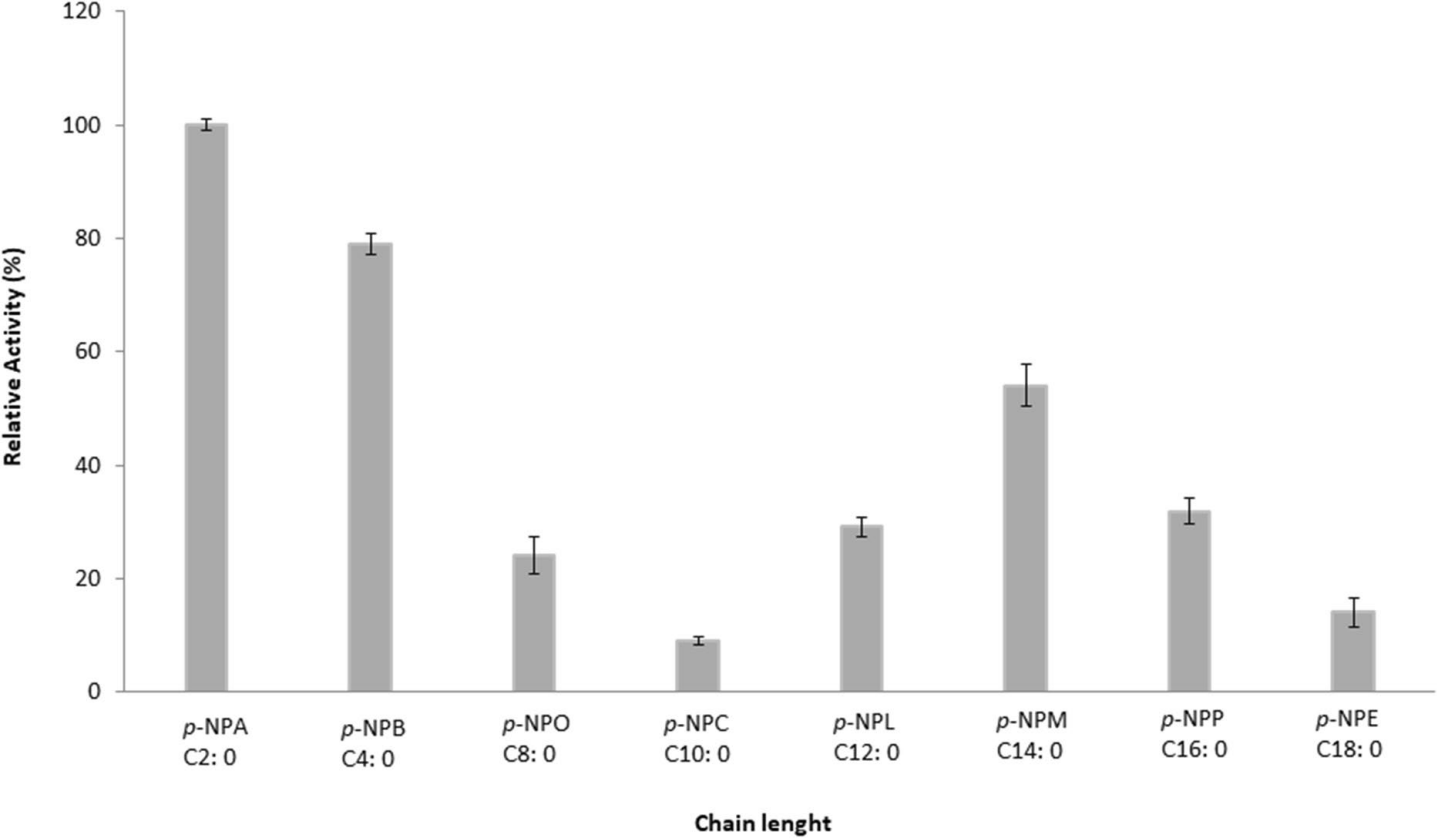
Substrate specificity of the enzyme ANCUT1 against *p*-NPE of different chain lengths. Relative activities were calculated using the specific activity of the control when p-NPA was used as substrate

ANCUT1 substrate preference was sought using different chain length esters using a wide range of *p*-NPE substrates with small chain lengths to long-chain lengths. Our results showed that ANCUT1 preferentially hydrolyzed short-chain length esters (C2–C4) and displayed a slight tendency for medium-chain length esters (C10–C14) (Fig. 10). Similar results have been observed for other cutinases, as these enzymes are intermediates between lipases and esterases (Maeda et al. 2005). Kinetic parameters of ANCUT1 using *p*-NPA as substrate are shown in Table 3.

**Table 3:** Kinetic parameters of ANCUT1

Kinetic parameter	Value
*V*max (mM/min)	58.82
*K*m (mM)	18.29
*K*cat (min^−1^)	220.49
*k*cat/*k*m (mM^−1^ min^−1^)	12.05
Catalytic cycle	0.28

We also tested the ability of ANCUT1 to degrade cutin, their natural substrate, by monitoring the generation of free fatty acids. However, it could not confirm the hydrolysis of cutin under our assay conditions. These results could be explained by differences in the size of the substrate-binding pocket; probably, ANCUT1 has a smaller binding pocket, but this must be corroborated. Besides, Bermúdez et al. (2019) found that ANCUT1 acts on fatty acids, forming cutin cross-linking bonds, which are more inaccessible and more challenging to identify using TLC assays.

An alternative test to cutin hydrolysis was performed to determine whether ANCUT1 can catalyze the hydrolysis of hydroxylated long-chain length esters. The propyl ester of ricinoleic acid was used, which is an 18-carbon fatty acid that has a hydroxyl group (C12) and a double bond (C9 ↔ C10) intermediate, like those found in the fatty hydroxy acids that make up cutin, with the difference of not having the hydroxyl group at the end of the fatty acid chain. Iodine-developed TLC plates showed a decrease in the intensity of the ricinoleate propyl (PRO) stain in reactions with ANCUT1 and lipase B from *C. antarctica* (CAL-B) between the first and third day of the reaction. In addition, some staining was observed at the bottom of the two reactions that could correspond to free fatty hydroxy acid to corroborate the hydrolysis of propyl ester (PM = 340 g/mol) and thus the release of ricinoleic acid (RIA) (PM = 298 g/mol).

**Fig. 11:**
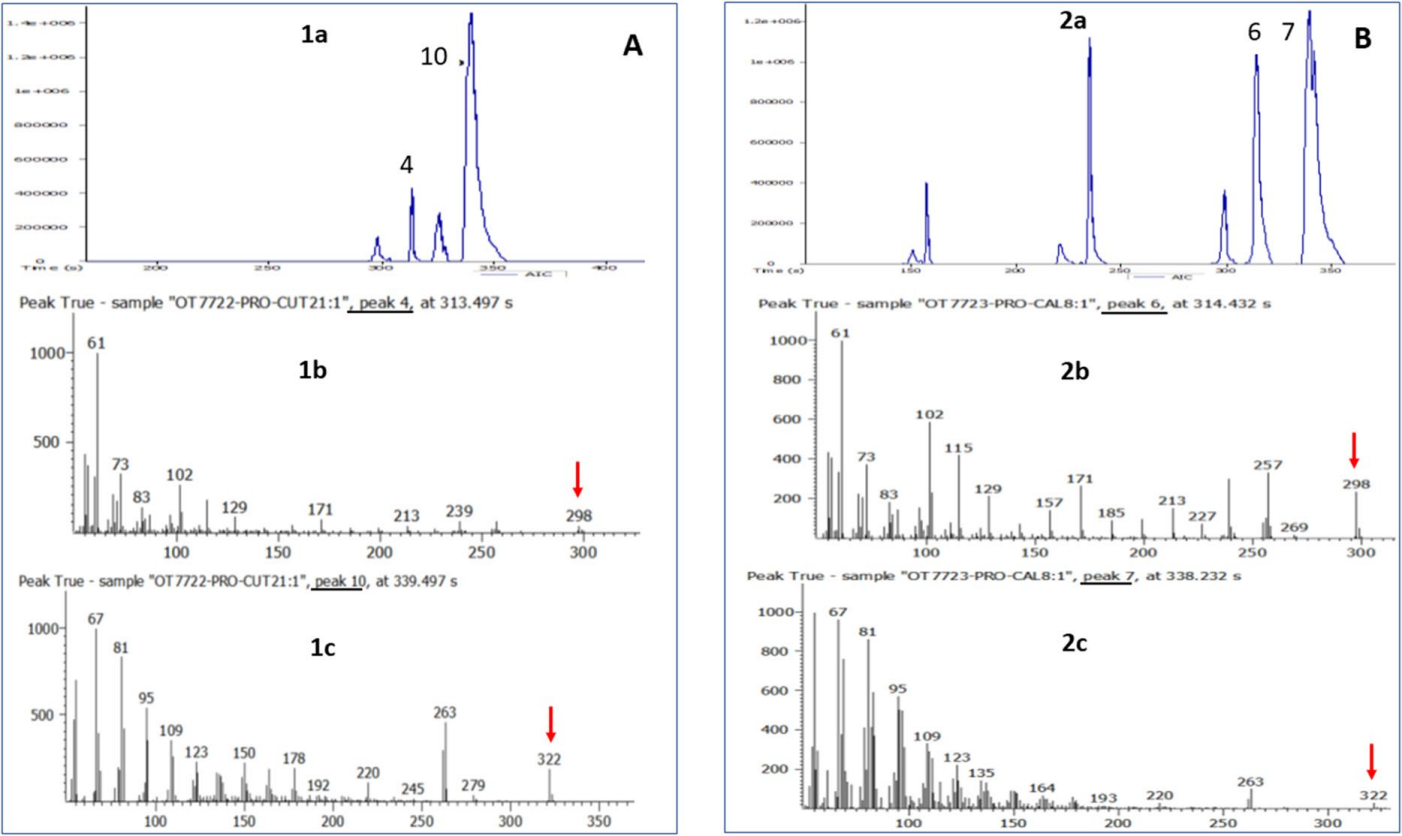
ESI-MS spectra of hydrolysis of propyl ricinoleate (PRO) with ANCUT1 (**A**) and CAL-B (**B**); (1a) PRO hydrolysis spectrum with ANCUT1; (1b) Ionisation pattern of ricinoleic acid (RIA), molecular ion m/z 298 is observed at 313 s; (1c) Ionisation pattern of PRO, ion m/z 322 is observed at 339 s; (2a) PRO hydrolysis spectrum with CAL-B; (2b) Ionisation pattern of RIA, the m/z 298 molecular ion is observed at 314 s; (2c) Ionisation pattern of PRO, the m/z 322 ion is observed at 338 s

ESI-MS spectrums of ANCUT1 and CAL-B reactions showed similar ionization patterns, where two prominent peaks after 290 s can be observed for both enzymatic reactions (Fig. 11). ANCUT1 spectrum has 4 at 313 and 10 at 339 s (Fig. 11, 1a), and CAL-B has 6 at 314 and 7 at 339 s (Fig. 11, 2a). The presence of ricinoleic acid as the molecular ion [M] m/z 298 was detected for both samples after analyses of peaks 4 and 6 (Fig. 11, 1b and 2b). Similarly, the presence of propyl ricinoleate (PRO) was noticed as the [M] m/z 322 on both samples, which corresponds to PRO molecular weight minus one molecule of water (340–18 = 322). Water absence is one of the typical losses that this molecular ion [M] suffers (Figs. 1c and 2c). Besides, this same m/z 322 ion was detected in the PRO standard spectrum (Supplementary material).

#### Effect of metal ions, inhibitors and surfactants

In Table 4, we observe that none of the metal ions had a considerable activating effect on ANCUT1, and many of the compounds decreased the cutinase activity by approximately 40% at 10 mM. Thus, we can conclude that the enzyme does not need a metal ion to potentiate its activity. Adding these salts to the medium decreased enzyme activity, possibly due to perturbations in the solvation layer of the enzyme. Other reports confirm this behavior and have shown that the activity of fungal cutinases decreases with the addition of metal ions (Duan et al. 2017). All the ions tested with ANCUT1showed a medium inhibitory effect, except for 1 mM Fe^3+^. The most considerable inhibitory effects were observed with Na^+^. To evaluate the requirement of metal ions for enzyme activity, we assayed activity in the presence of two concentrations (1 mM and 10 mM) of the metal-chelating agent EDTA. The activity of ANCUT1 decreased in the presence of both concentrations of EDTA (64.4% and 43.6%, respectively), while the previously reported cutinase, ANCUT2, was only affected by 10 mM EDTA (Bermúdez-García et al. 2017).

**Table 4:** Effect of metal ions and surfactants on the activity of ANCUT1

Enzyme	Residual activity (%)	
Compound	Concentration	
	1 mM	10 mM
EDTA	64.425 ± 1.72	43.599 ± 2.59
Ca^2+^	89.239 ± 0.35	45.455 ± 1.38
Mg^2+^	71.011 ± 5.87	50.093 ± 4.67
K^+^	68.460 ± 4.16	45.269 ± 4.16
Na^+^	74.397 ± 2.77	38.776 ± 3.89
Fe^3+^	102.041 ± 4.32	52.412 ± 1.96
Cu^2+^	87.570 ± 2.77	51.299 ± 2.13
PMSF	36.642 ± 3.45	35.065 ± 0.69
SDS	55.659 ± 4.15	23.191 ± 6.92
Tween 80	45.501 ± 2.36	35.993 ± 5.87

Enzyme activity was evaluated in the presence of nonionic and anionic surfactants. In the case of Tween 80, the enzymatic activity of ANCUT1 was reduced by about 55% of the initial value at a concentration of 1 mM; when a higher concentration (10 mM) was used, ANCUT1 lost 64%. A cutinase of *T. fusca* also loses nearly all its activity after adding nonionic surfactants; in contrast, other cutinases of *T. fusca* and *F. solani* are unaffected (Duan et al. 2017). Plou and coworkers demonstrated that Tween 80 could act as a substrate for esterases/lipases and, therefore, be a competitive inhibitor of *p*-NFE (Plou 1998). SDS has been reported to inhibit cutinases by causing conformational changes in the active site (Pocalyko and Tallman 1998). The possible inhibitors decreased the enzyme activity by more than 60% in the two concentrations tested by decreasing the surface tension. PMSF is a serin-protease inhibitor; cutinases also have a catalytic serin that PMSF can inhibit. As expected, PMSF has an inhibitory effect in ANCUT1, although this was not detrimental; this has also been observed in other cutinases (Altammar et al. 2022).

Moreover, adding anionic detergents such as SDS could result in protein denaturation by disrupting the hydrophobic interactions between the protein residues and causing aggregation (Plou et al. 1998). In this report, we found that 10 mM SDS strongly affected the activity of ANCUT1, causing an 80% loss.

#### Effect of different solvents

It is advantageous to define the stability of the enzymes in different solvents, as many industrial organic reactions occur in the presence of solvents (Dutta et al. 2009). In this sense, it is well recognized that most enzymes are more stable in hydrophobic solvents, potentially because they can dissolve proteins, resulting in the loss of their tertiary structure (Chin et al. 1994). The results of these experimentations are presented in Table 5. ANCUT1 was more stable than the previous cutinase characterized in our group (ANCUT2) in the presence of all the solvents tested (Bermúdez-García et al. 2017), and it is also more stable than other evaluated cutinases (Chen et al. 2010; Speranza et al. 2011), displaying high stability in all solvents tested, showing the highest in hexane followed by DMSO.

**Table 5:** Effect of solvents in the activity of ANCUT1

Residual activity (%)		
Solvent	30%	50%
Acetone	81.379 ± 1.58	79.540 ± 2.24
Ethanol	88.736 ± 3.11	74.483 ± 2.97
DMSO	96.782 ± 3.46	80.690 ± 3.70
Isopropyl alcohol	95.862 ± 2.44	78.008 ± 2.69
Hexane	105.287 ± 1.38	86.897 ± 1.90

### Transesterification of methyl-coumarate to butyl coumarate

**Fig. 12:**
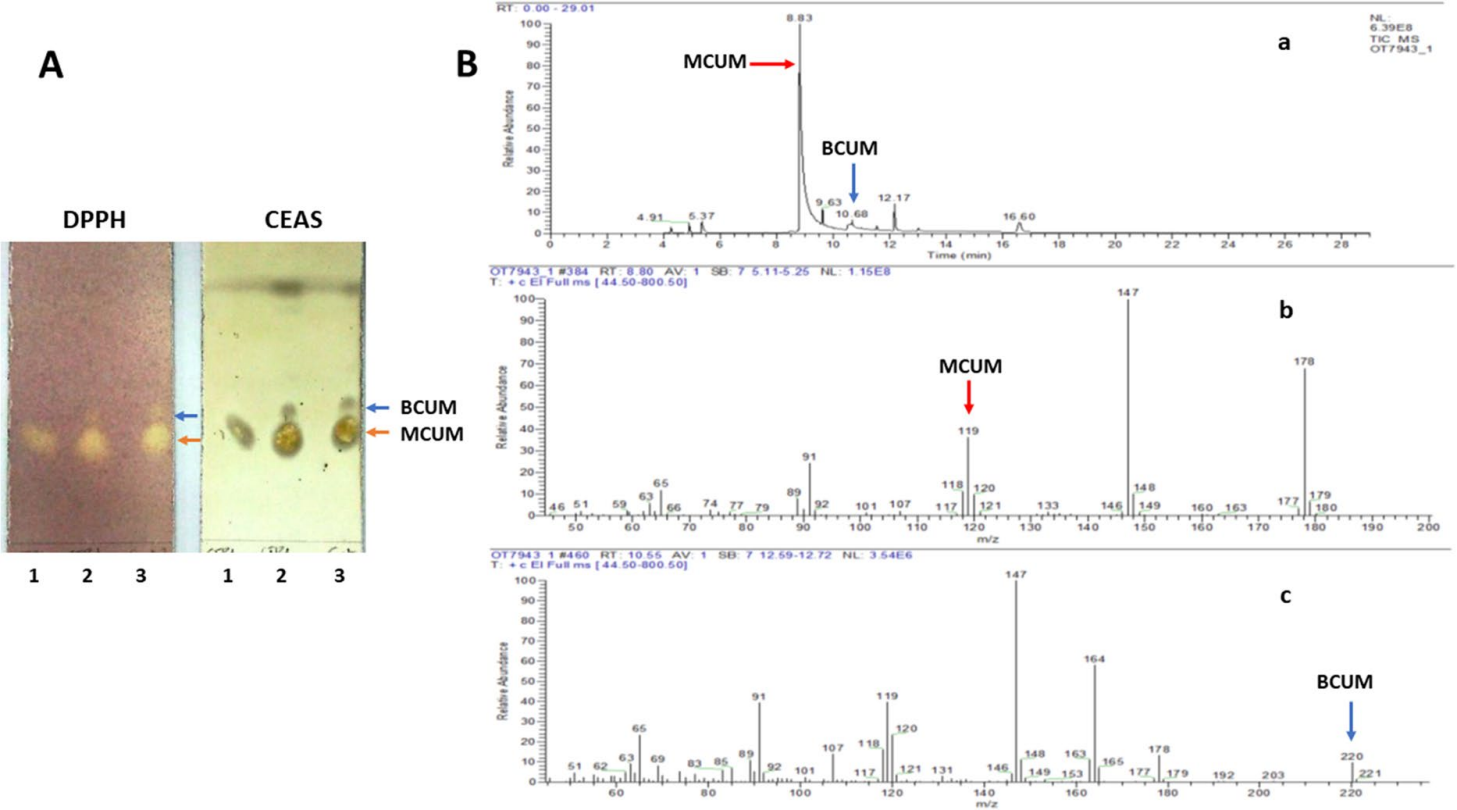
Transesterification of methyl coumarate (MCUM) to butyl coumarate (BCUM). Panel **A**, Products of transesterification on TLC stained with DPPH and CEAS. Lane 1: Standard MCUM used as control (CTRL); lane 2: CTRL and products of enzymatic transesterification reaction with ANCUT1; lane 3: Products of enzymatic transesterification reaction with ANCUT1. The MCUM (substrate) and BCUM (the expected product) are signaled with blue and red arrows. Panel **B**, ESI–MS spectra of MCUM to BCUM transesterification reaction. (a) Spectrum of the enzymatic transesterification reaction, at 8.83 min, there is the base peak belonging to MCUM, and at 10.68 min there is the peak belonging to BCUM signaled with blue and red arrows; (b) Ionization pattern of MCUM, the molecular ion m/z 178 is marked with a red arrow; (c) Ionization pattern of BCUM, the molecular ion m/z 220 is marked with a blue arrow

Methyl coumarate (MCUM) lipophilization catalyzed by ANCUT1 through a transesterification reaction with butanol to obtain butyl coumarate (BCUM) was tested. The BCUM production was visualized in TLC plates (Fig. 12, panel A). The MCUM control and the reaction product (BCUM) were analyzed by ESI–MS (Fig. 12, panel B). In the spectrum of the reaction, the base peak was observed at 8.83 min (Fig. 12, panel Ba), which corresponds to MCUM (PM = 178 g/mol) by comparison of its ionization pattern with the standard spectrum of MCUM at 10.55 min (Fig. 12, panel Bb). A similar ionization pattern to that of MCUM was observed, but the molecular ion m/z 220 also corresponded to BCUM (PM = 220 g/mol) (Fig. 12, panel B c). The relative abundance of MCUM (100%) and BCUM (5%) seen in the reaction spectrum (at 8.83 and 10. 68 min, respectively) agrees with what was observed in the TLC plates, where MCUM remained the compound of highest abundance after the five days of reaction. Only a tiny part of it was converted to the compound of higher hydrophobicity, BCUM. However, it is relevant to point out that few reports on the enzymatic synthesis of alkyl coumarates exist. Besides, ANCUT1 can carry out this reaction, typically reported with lipases or feruloyl esterases (Sharma et al. 2014; Vega-Rodríguez 2021). Optimization of reaction parameters must be done to improve this yield.

## Conclusions

A new thermo-alkaline cutinase (ANCUT1) from *A. nidulans* was characterized. The enzyme was better obtained by adding apple cutin (0.4%) and glycerol (0.5%) to a minimal medium. ANCUT1 has better stability than the previously described ANCUT2 at various temperatures and under the presence of solvents, surfactants, and metal ions, probably because of the presence of disulfide bonds, saline bridges and prolines un loop regions. Additionally, their substrate specificity is different. Considering the obtained properties of thermo-alkalinity, versatile hydrolytic activity, tolerance to surfactants, and superior stability in organic solvents, ANCUT1 displays properties that may make it useful for many industrial applications. An interesting chemical reaction, such as the transesterification of hydroxycinnamic acids, was demonstrated.

### Supplementary Information

Below is the link to the electronic supplementary material.

Supplementary file1 (TIF 159 KB)
