# Supplementary figures and images for: ANCUT1, a novel thermoalkaline cutinase from Aspergillus nidulans and its application on hydroxycinnamic acids lipophilization
Source: Biotechnol Lett. 2024 Feb 28;46(3):409–30. doi: 10.1007/s10529-024-03467-2 (PMC11055803; doi:10.1007/s10529-024-03467-2)

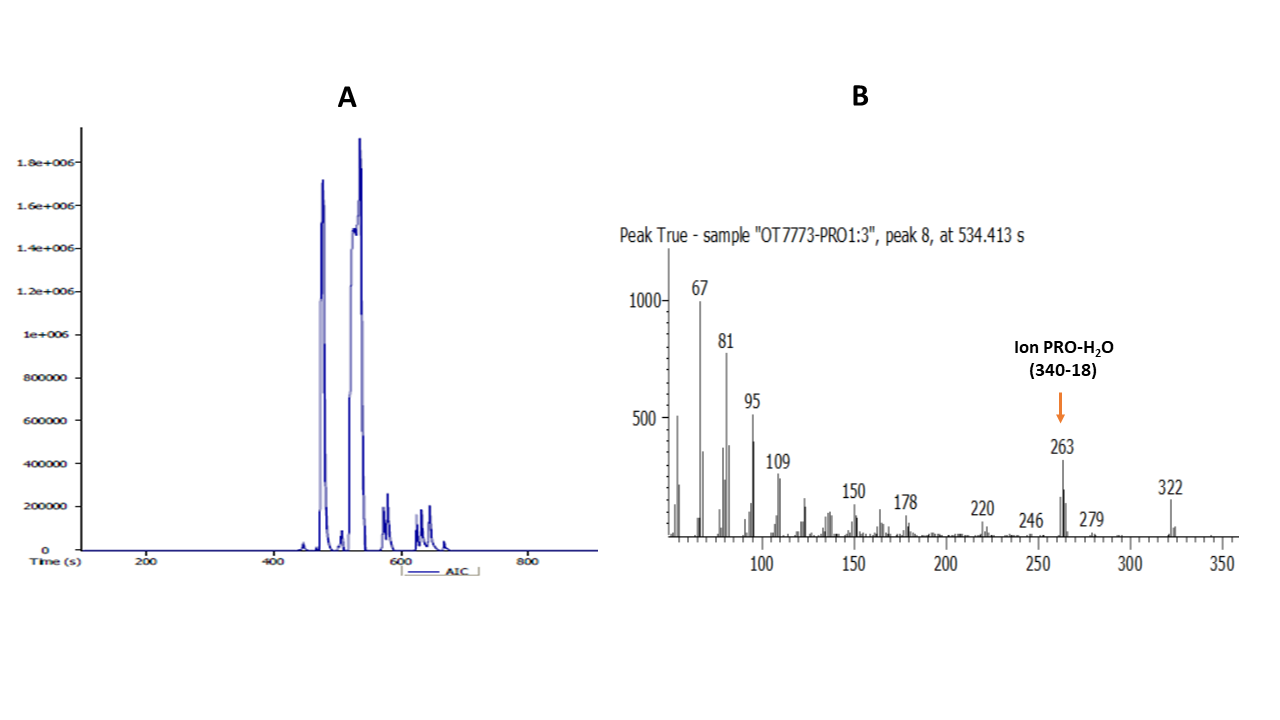

Supplement: Supplementary file 1 — Supplementary file1 (TIF 159 KB) [file 10529_2024_3467_MOESM1_ESM.tif]
